# The role of carbohydrate drinks in preoperative nutrition: comment 1

**DOI:** 10.1308/003588413X13511609956453

**Published:** 2013-01

**Authors:** D Leaper

**Affiliations:** Cardiff University,UK

**Comment on**

**Jones C, Badger SA, Hannon R.** The role of carbohydrate drinks in preoperative nutrition for elective colorectal surgery. *Ann R Coll Surg Engl* 2011; **93**: 504–507

doi 10.1308/147870811X13137608455136

I enjoyed reading and entirely agree with the findings of the review by Jones *et al* on the role of carbohydrate (CHO) drinks in preoperative nutrition for elective colorectal surgery but I offer a word of caution. No mention was made of the possible increased risk of surgical site infection (SSI) that may be caused by this action, even in non-diabetic patients. Tight control of perioperative blood sugar in patients who have diabetes is one of the high impact interventions advocated by the Department of Health to reduce the risk of SSI.^1^ In addition, there is compelling evidence that poorly controlled, perioperative blood sugar, even in non-diabetic patients, may significantly increase deep sternal wound SSI rates in patients having cardiac surgery.^2^

In these days of enhanced recovery after surgery (ERAS), the increasing use of a laparoscopic approach (particularly for elective colorectal surgery) and optimal use of anaesthesia^3^ (both of which minimise the metabolic response to trauma) may make this caution unnecessary with regard to SSI. However, I should be interested to know if Jones *et al* found any increased incidence of SSI in their meta-analysis when preoperative CHO drinks had been used prior to open elective colorectal surgery. If this were found to be the case, closer control of blood sugar ought to be considered in the perioperative period.
